# Inhibition of Inflammation, Suppression of Matrix Metalloproteinases, Induction of Neurogenesis, and Antioxidant Property Make Bryostatin-1 a Therapeutic Choice for Multiple Sclerosis

**DOI:** 10.3389/fphar.2018.00625

**Published:** 2018-06-19

**Authors:** Fahimeh Safaeinejad, Soheyl Bahrami, Heinz Redl, Hassan Niknejad

**Affiliations:** ^1^Department of Pharmacology, School of Medicine, Shahid Beheshti University of Medical Sciences, Tehran, Iran; ^2^Ludwig Boltzmann Institute for Experimental and Clinical Traumatology, AUVA Research Centre, Vienna, Austria

**Keywords:** multiple sclerosis, Bryostatin-1, matrix metalloproteinases, anti-inflammation, neuroprotection, neurogenesis

## Abstract

Multiple sclerosis (MS) is a neurodegenerative disease characterized by inflammation and myelin damage. Pro-inflammatory cytokines, oxidative stress, high level of matrix metalloproteinases (MMPs) activity and blood–brain barrier (BBB) damage, immune-mediated destruction of myelin and neuron loss are involved in the pathogenesis of MS. The currently approved treatments for MS include injectable drugs (interferon-β and glatiramer acetate), oral drugs (fingolimod), and monoclonal antibodies (natalizumab). The mentioned therapeutic choices are mostly focused on the inhibition of inflammation. Therefore, the search for a multi-target therapeutic choice remains unchallenged. It seems that a drug with anti-inflammatory, oxidative stress inhibitory, reduction of MMPs activity, and neurogenesis stimulatory properties may be effective for treatment of MS. In this regard, Bryostatin-1 as a macrolide and marine natural product has been selected as a therapeutic choice. Studies indicate that Bryostatin-1 has anti-inflammatory and antioxidant properties and decreases MMPs level and BBB damage. Furthermore, Bryostatin-1 has a neuroprotective effect and promotes neurogenesis and differentiation of oligodendrocyte progenitor stem cells as a critical step for remyelination/myelogenesis. Based on these properties, we hypothesized here that Bryostatin-1 is an effective treatment in MS.

## Introduction

Multiple sclerosis (MS) is a neurodegenerative disease, characterized by inflammation and demyelination. It is the second common reason for disability in young adults. MS affects three folds more in women than men (Wingerchuk and Carter, 2014). It often occurs in high-income countries with a heterogeneous prevalence worldwide (over 2,000,000 people around the world). MS has the highest prevalence in North America (140/100,000 population) and Europe (108/100,000), and the lowest in East Asia (2.2/100,000 population) and sub-Saharan Africa (2.1/100,000). So, the majority of countries encounter MS as a disabling disease (Naegele and Martin, 2014; Leray et al., 2016).

Various environmental (geographical, infectious, and/or nutritional) and genetic factors are involved in MS. Therefore, it is recognized as a multifactorial disease (Esposito et al., 2017). Pathogenesis of the disease is not well known but the hallmark of MS is lesions caused by infiltration of immune cells from the blood–brain barrier (BBB), and then enhances inflammation, demyelination, and neurodegeneration in the central nervous system.

The inflammatory process involves different immune cells such as Th1, Th2, macrophage, and B cells (Naegele and Martin, 2014; Tehrani et al., 2017). Since inflammation is considered as a main cause of MS, anti-inflammatory, and immunomodulatory drugs are among the primary therapeutic choices. Approved anti-inflammatory treatments for MS include injectable therapies [interferon-β (IFN-β) and glatiramer acetate], oral therapies (e.g., teriflunomide, dimethyl fumarate, and fingolimod), and infusion therapies (mitoxantrone, natalizumab, and alemtuzumab) (Mallucci et al., 2015).

Another possible cause of pathogenesis is oxidative stress. Mitochondrial DNA and proteins are extremely vulnerable to oxidative damage. Mitochondrial changes are the reason for oligodendrocytes apoptosis in MS (Niknejad et al., 2016; Yun et al., 2017). Therefore, antioxidant treatments can protect oligodendrocytes and neurons.

The role of matrix metalloproteinases (MMPs) has been extensively investigated in the pathogenesis of MS. The studies revealed that MMPs (types II, VII, IX, and XII) secreted from leukocytes, microglia, neurons, and reactive astrocytes digest myelin basic protein which lead to axonal demyelination, neuronal cell death, and MS progression. In addition, MMPs are the main cause of disrupting the BBB integrity which facilitates immune cells migration into the brain. Therefore, MMP inhibitors can contribute to the improvement of MS. Some synthetic inhibitors and drugs such as minocycline and D-penicillamine have been investigated in this regard so far (Rempe et al., 2016).

Lastly, neuronal damage in MS lesions has been reported in neuropathological research. Mechanisms causing myelin destruction are not entirely known. Typically, MS lesions consist of the BBB breakdown, multifocal inflammation, demyelination, oligodendrocyte damage, axonal degeneration, and neuron loss. This damage is likely to be a result of inflammation in the CNS (Dutta and Trapp, 2011). Therefore, the protection of neurons from possible damage and inducing neurogenesis and myelogenesis in the CNS are therapeutic targets which overcome the MS challenges.

Altogether, it seems that a drug with anti-inflammatory, oxidative stress inhibitory, MMPs inhibitory, and neurogenesis stimulatory properties may be effective for the treatment of MS. There is some evidence which Bryostatin-1 (a macrolide and marine natural product) is a therapeutic choice for MS.

## Supporting Evidences of Hypothesis

### Evidence 1: Anti-inflammation

T helper cells are one of the important components of the immune system. T helper cells have various phenotypes include Th1, Th2, Th17, and T_regs_; each one has its specific roles. Th1 participates in inflammatory processes. In MS, the early event in the initiation of inflammation in the brain is the migration of activated T cells through the BBB. Activated Th1 cells stimulate cytokines such as TNF-α, IFN-γ, and IL-2 which are expressed in MS lesions. Th2 is involved in anti-inflammatory processes by different cytokines production such as IL-5 and IL-10 (Keelan et al., 2003).

*In vivo* experiments have shown that Bryostatin-1 is able to enhance Th2 responses and significantly increase Th2 cytokines levels such as IL-5 and IL-10. So, Bryostatin-1 shifts the immune response toward Th2 type profile which is associated with immunological tolerance (Ariza et al., 2011). One of the most known mechanisms in inflammatory reactions is up-regulation of cyclooxygenase-2 (COX-2). It has been recently shown that treatment with Bryostatin-1 can sharply down-regulate the mRNA expression level of COX-2 (Salim et al., 2018).

Kornberg et al. (2018) recently showed that Bryostatin-1 enhances an anti-inflammatory phenotype in macrophages and APCs. They observed that Bryostatin-1 up-regulates CD86 (a costimulatory protein required for activation of T cells upon their binding to APCs) in bone marrow-derived dendritic cells (BMDCs), whereas it has no significant effect on CD40 (a costimulatory protein necessary for pro-inflammatory activation of APCs). Moreover, Bryostatin-1 treatment of murine peritoneal macrophages promotes the IL-4-induced expression of arginase-1, a marker of the anti-inflammatory and repair-promoting M2 macrophage phenotype. Similar to its effect on dendritic cells, low-dose of Bryostatin-1 inhibits secretion of proinflammatory cytokines such as IL-12 and IL-6. In contrast, Bryostatin-1 considerably increases secretion of IL-10, an anti-inflammatory cytokine that inhibits Th1 differentiation and stimulates an immunoregulatory environment (Kornberg et al., 2018).

Also, a higher level of TNF-α released by stimulation of Th1 cells causes more BBB damage (Kornek and Lassmann, 2003). Dental et al. (2017) found that treatment with Bryostatin-1 leads to a significant decrease of BBB permeability caused by TNF-α.

### Evidence 2: Anti-oxidant Property

The production of free radicals like hydroxyl radical (-OH) and hydrogen peroxide (H_2_O_2_) is one of the causes of cellular damage. If the antioxidant defense does not have the capability of scavenging free radicals, they induce cell death in neural cells (Niknejad et al., 2012a).

Shang et al. (2016) studied the anti-oxidant effect of Bryostatin-1. Their results revealed that pre-treatment of cultured cells with Bryostatin-1 inhibit H_2_O_2_ induced cell death. Caspases after activation by mitochondria play an important role in the regulation of cell apoptosis. Bryostatin-1 can inhibit apoptosis in some cells via a reduction in the activation of caspase-3 and -9. Bcl-2 is another factor involved in the regulation of apoptosis by mitochondrial pathway. Bryostatin-1 is able to increase the expression level of Bcl-2 as an anti-apoptotic factor (Shang et al., 2016). In a study conducted by Sun and Alkon (2008), there was a synergistic effect between Bryostatin-1 and α-tocopherol to reduce oxidative stress damage in neural cells.

### Evidence 3: Suppression of Matrix Metalloproteinases Expression

Matrix metalloproteinases (MMPs) are involved in extracellular matrix (ECM) degradation, cell apoptosis, and degradation of the basement membrane (Niknejad et al., 2012b; Niknejad and Yazdanpanah, 2014). MMPs cause damage to the brain in MS through two mechanisms: first, MMPs produced by leukocytes degrade vessel endothelial lining and result in BBB breakdown and the entrance of inflammatory cells to CNS. Second, MMPs can damage the myelin sheath and cause more demyelination and inflammation into the CNS (Javaid et al., 2013). Treatment with Bryostatin-1 suppresses production of MMP-1, -3, -9, -10, and 11 (Kortmansky and Schwartz, 2003; Ruiz-Torres et al., 2017). Also, it has been reported that Bryostatin-1 reduces the hemorrhagic transformation following ischemic/reperfusion injuries and improves BBB disruption by down-regulation of MMP-9 expression and activation (Tan et al., 2015). Sun et al. (2016) reported that Bryostatin-1 is noticeably able to suppress the expression of MMP-2 and MMP-9 in a breast cancer cell line.

### Evidence 4: Neuroprotective Effects

Tan et al. (2013) showed that Bryostatin-1 is able to attenuate both necrotic and apoptotic cell death of neurons in ischemic brain injury. Furthermore, Bryostatin-1 ameliorates neurological functions and decreases inflammatory responses. Therefore, it is a neuroprotective agent (Tan et al., 2013). In addition to inhibition of cell death, Bryostatin-1 neuroprotective effects may be due to the increase of the neurotrophin production in neural cells (Nelson and Alkon, 2009).

By studying an animal model of traumatic brain injury (TBI), Lucke-Wold et al. (2015) found that Bryostatin-1 decreases toxic PKCα levels and increases neuroprotective isozyme PKC𝜀. Also, because of PKC isozymes modulation, it reduces BBB breakdown by a notable rise in the tight junction proteins including VE-cadherin, ZO-1, and occluding (Lallemend et al., 2005; Lucke-Wold et al., 2015). It has been revealed that Bryostatin-1 is able to induce neurite regrowth of spiral ganglion neurons (SGNs) by PKCβI activation mechanism (Lallemend et al., 2005). Moreover, Bryostatin shows a neuroprotective effect on auditory neurons (Poirrier et al., 2013).

The elevated α-Synuclein expression in oligodendrocytes has been shown in an animal model of MS. The abnormal α-Synuclein accumulation in cells probably contributes to oligodendrocytes death in MS. Moreover, Lu et al. (2009) demonstrated that α-Synuclein is present in the active inflammatory lesions of MS. It has been shown that the treatment with Bryostatin-1 causes a significant reduction in α-synuclein expression (Sun et al., 2016).

### Evidence 5: Neurogenesis

Recent studies make evident that the administration of Bryostatin-1 after global ischemic insult in the brain results in curative neurogenesis, synaptogenesis, and cognitive improvement (Tan et al., 2013).

In an animal model, Sun et al. (2008) showed that Bryostatin-1 administration improves deficits in neurotrophic activity and synaptogenesis after ischemia. Bryostatin-1 ameliorates learning and memory by preventing neuronal damage and restoring the dendritic spines and their synapses (Sun et al., 2008). Bryostatin-1 promotes exercise-dependent paralysis recovery in animals with stroke. It is possibly related to the synaptic conduction efficiency and synaptic plasticity (Mizutani et al., 2015). Therefore, Bryostatin-1 has neurogenesis and synaptogenic effects which can overcome memory impairment as a common problem in MS (DeLuca et al., 2013).

### Evidence 6: Differentiation of OPCs as a Critical Step for Remyelination/Myelogenesis

Previous results showed that myelin proteins, which accumulate following demyelination, inhibit remyelination by inhibition of oligodendrocyte progenitor cells (OPCs) differentiation into myelinating oligodendrocyte via PKCα modulation. Bryostatin-1 has been shown to overcome the inhibitory effects of myelin protein extracts on OPC differentiation and induce direct differentiation of oligodendrocyte progenitor stem cells into myelinating oligodendrocyte via inhibition of PKCα (Gonzalez et al., 2016).

## Hypothesis

**FIGURE 1:**
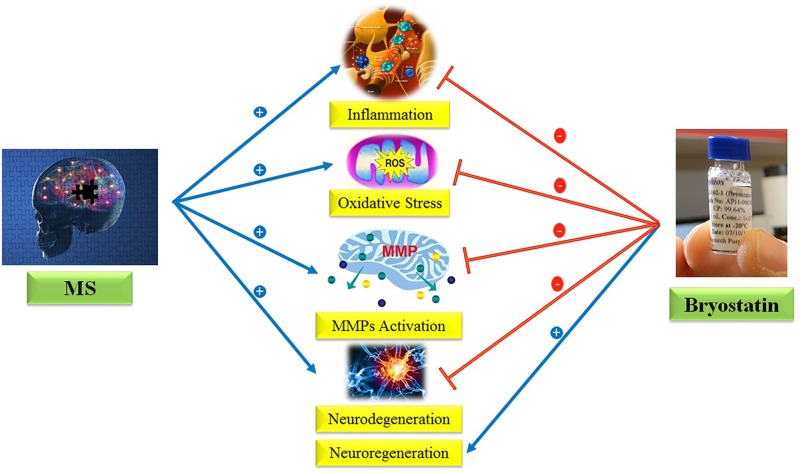
Anti-inflammatory, anti-oxidant, neuroprotective, neurogenesis, and inhibition of MMPs properties of Bryostatin-1.

Regarding the provided evidences, we hypothesize here that Bryostatin-1 could be used to treat MS due to its anti-inflammatory, anti-oxidant, neuroprotective, neurogenesis, and inhibition of metalloproteinase matrix properties (**Figure 1**).

### Evaluation of Hypothesis

To evaluate the hypothesis, it is necessary to do some *in vitro* and *in vivo* experimental studies which can address the building block questions of the hypothesis.

#### *In Vitro* Studies

To evaluate the neuroprotective and anti-oxidant properties of Bryostatin-1, oligodendrocyte cell line such as the OLN-93 will be cultured and oxidative stress and cell death will be induced by H_2_O_2_ or cuprizone. The viability tests (and qualitative and quantitative analysis of apoptotic markers) and the measurement of neurotrophin levels and mitochondrial assays before and after treatment with Bryostatin-1 give us some useful information about the effects of Bryostatin-1 on the mechanisms involved in MS such as oxidative stress.

#### *In Vivo* Studies

To evaluate the *in vivo* effects of Bryostatin-1, the toxic model of MS is created in C57BL/6 mice by cuprizone. In order to measure the motor coordination and balance, the behavioral test of rotarod will be carried out before and after treatment with Bryostatin-1. Cellular and molecular tests using immunohistochemistry, Western blot, and real time-PCR analyses on the myelin genes and proteins and microscopic examinations will help us to investigate the effects of Bryostatin-1. To determine the reduction of inflammation by Bryostatin-1, inflammatory, and anti-inflammatory cytokines are measured before and after treatment with Bryostatin-1 via Elisa kits. Also, the effect of Bryostatin-1 on the suppression of the MMPs will be evaluated on the expression levels. Finally, to evaluate the effects of Bryostatin on OPC differentiation and remyelination, oligodendrocyte precursor marker (Olig2) and adult myelin markers (PLP, MBP, and MOG) is examined by real time-PCR and Western blot analyses.

## Discussion

Multiple sclerosis is still a challenging disease without a curative treatment (Torkildsen et al., 2016). Due to the fact that MS is a complex and multifactorial disease (Esposito et al., 2017), treatment must be multi-purpose and target simultaneously several pathological features of MS. Currently, the available medications have more effects on inflammatory aspects of MS. We suggested here that Bryostatin-1 has enough criteria to be as a choice for the MS therapy. Since it has anti-inflammatory, anti-oxidant, MMP inhibitory, neuroprotective, and neurogenic properties, it can cover many aspects of the pathogenesis of MS. Bryostatin-1 has been positively used in various animal models and clinical trials of neural injury, including auditory neurodegeneration, ischemic stroke, TBI, and Alzheimer’s disease. After global ischemic insult, Bryostatin-1 administration resulted in improvement of cognition, synaptogenesis, and neurogenesis, and decline of necrosis and astrogliosis in infarcted tissue. Moreover, it has direct antitumor effects in lymphoma, sarcoma, leukemia, melanoma, and is used breast, colon, lung, and ovarian cancers (Enna and Bylund, 2009; Tabatabaei Rezaei et al., 2015). In addition, it has several pharmacological actions including functional restoration and cognitive improvement in dementia and depression, a decline in the accumulation of neurotoxic amyloid and an inhibition of tau protein hyper-phosphorylation. Based on the data accessible in the preclinical and clinical studies, pharmacological and toxicological aspects of Bryostatin-1 have been demonstrated. Bryostatin-1 is well tolerated and its adverse reactions are rare, mild, and reversible. A major advantage of this drug is that it can be administered orally. Furthermore, its biological and elimination half-life is suitable. Today, the synthetic form of Bryostatin-1 is available which is cost-effective. Bryostatin-1 can easily cross the BBB, which is an advantage for treating MS in low doses (Sun and Alkon, 2006).

In the end, to the best of our knowledge, the majority of studies about the effects of Bryostatin-1 are on the cellular level. Therefore, it is necessary to focus on the molecular events occurred after treatment of the cells with Bryostatin-1 in the future studies, especially in the field of evidences mentioned in this paper such as oxidative stress, inflammation, myelogenesis and so on.

## Conclusion

In conclusion, Bryostatin-1 with anti-inflammatory, anti-oxidant, MMPs inhibitory, and neurogenesis stimulatory properties and other advantages include the ability to cross the BBB and oral availability may be appropriate for the treatment of MS.

## Author Contributions

FS and HN hypothesized the idea, wrote the manuscript and revised it. SB and HR revised the manuscript.

## Conflict of Interest Statement

The authors declare that the research was conducted in the absence of any commercial or financial relationships that could be construed as a potential conflict of interest. The reviewer AM and handling Editor declared their shared affiliation.
